# Pharmacological Inhibition of ULK1 Kinase Blocks Mammalian Target of Rapamycin (mTOR)-dependent Autophagy*[Fn FN2]

**DOI:** 10.1074/jbc.C114.627778

**Published:** 2015-04-01

**Authors:** Katy J. Petherick, Owen J. L. Conway, Chido Mpamhanga, Simon A. Osborne, Ahmad Kamal, Barbara Saxty, Ian G. Ganley

**Affiliations:** From the ‡ Medical Research Council (MRC) Protein Phosphorylation and Ubiquitylation Unit, College of Life Sciences, University of Dundee, Dundee DD1 5EH, Scotland and; the § MRC Technology Centre for Therapeutics Discovery, 1-3 Burtonhole Lane, Mill Hill, London NW7 1AD, United Kingdom

**Keywords:** autophagy, cell biology, enzyme inhibitor, protein kinase, signal transduction, ULK1, cancer

## Abstract

Autophagy is a cell-protective and degradative process that recycles damaged and long-lived cellular components. Cancer cells are thought to take advantage of autophagy to help them to cope with the stress of tumorigenesis; thus targeting autophagy is an attractive therapeutic approach. However, there are currently no specific inhibitors of autophagy. ULK1, a serine/threonine protein kinase, is essential for the initial stages of autophagy, and here we report that two compounds, MRT67307 and MRT68921, potently inhibit ULK1 and ULK2 *in vitro* and block autophagy in cells. Using a drug-resistant ULK1 mutant, we show that the autophagy-inhibiting capacity of the compounds is specifically through ULK1. ULK1 inhibition results in accumulation of stalled early autophagosomal structures, indicating a role for ULK1 in the maturation of autophagosomes as well as initiation.

## Teaser

**Background:** Autophagy is an intracellular lysosomal degradation pathway implicated in many diseases, but there are currently no specific autophagy inhibitors.

**Results:** Small molecule inhibition of ULK1, the upstream autophagy initiating kinase, blocks autophagosome initiation and maturation.

**Conclusion:** ULK1 plays a role in autophagosome maturation as well as initiation.

**Significance:** ULK1 can be targeted to block autophagy for disease therapy.

## Introduction

Macroautophagy, called autophagy in this instance, is a membrane-driven process that traffics intracellular components to the lysosome for recycling (1). It is essentially a cell survival mechanism and is up-regulated under conditions of stress, allowing cells to supply building blocks and energy to maintain function during periods of starvation. Autophagy also eliminates impaired or foreign components that if left to persist could cause damage. Due to these functions, autophagy has been linked to many diseases from neurodegeneration to cancer (2). It is therefore an attractive therapeutic target, especially in cancer where it acts as a survival mechanism allowing the tumor to cope with an increased metabolic demand and damage caused by chemotherapeutics (3, 4). Hence, autophagy inhibition is thought to be a viable treatment approach. However, to date there are no specific small molecule autophagy inhibitors, and this has hampered validation of autophagy as a potential target in cancer. Hydroxychloroquine has entered clinical trials as an autophagy inhibitor (5–11), but it is far from specific given that it disrupts lysosomal function. The lysosome is the end point of autophagy as well as most other intracellular transport pathways including endocytosis.

The most understood pathway of autophagy activation occurs upon inhibition of mTOR,^2^ either by amino acid withdrawal or by a direct pharmacological block. This leads to activation of the ULK1 protein kinase complex followed by the VPS34 lipid kinase complex (12–15). This in turn leads to the recruitment of a unique ubiquitin-like conjugation system resulting in the conjugation of LC3, and its family members, to the lipid phosphatidylethanolamine in the forming autophagosome, or phagophore. This lipidated form of LC3, termed LC3-II, is thought to drive elongation and cargo incorporation into the autophagosome (16). Thus there are many potential targets to inhibit autophagy. Kinase inhibitors have proved successful in the clinic, making ULK1 and VPS34 attractive candidates for inhibition. Studies about specific VPS34 inhibitors have recently been published, and these substances do indeed inhibit autophagy (17–19). However, VPS34 is also essential for sorting in the endocytic pathway, meaning that like hydroxychloroquine, VPS34 inhibition also affects endocytosis and lysosomal turnover in general. ULK1, on the other hand, is believed to be specific for autophagy, although other roles cannot be ruled out at this stage (20). We therefore set out to identify compounds that could inhibit ULK1 activity, not only to act as tool reagents to aid research but importantly to provide a proof-of-principle that targeting ULK1 could block autophagy.

## EXPERIMENTAL PROCEDURES

### 

#### 

##### Reagents and Antibodies

MRT compounds were synthesized as described (21). AZD8055 was from Selleck Chemicals, and bafilomycin A1 was from Enzo Life Sciences. Rabbit anti-ULK1, anti-ATG5, and anti-ATG13 were from Sigma. Rabbit anti-phospho-Ser-757 ULK was from Cell Signaling Technology, and rabbit anti-phospho-Ser-318 ATG13 was from Abnova. Mouse anti α-tubulin was from Merck-Millipore. Mouse anti-LC3 for immunofluorescence was from MBL International, and sheep anti-LC3, used for immunoblotting, was generated by the Division of Signal Transduction Therapy, University of Dundee, from recombinant full-length human GST-LC3b and affinity-purified.

##### Recombinant DNA

Procedures were performed using standard protocols and mutagenesis was performed using QuikChange site-directed mutagenesis (Stratagene). Constructs are described and available on the MRC-PPU Reagents website.

##### Cell Culture

Immortalized wild-type mouse embryonic fibroblasts (MEFs) have been described previously (22). LKB1 MEFs were a kind gift from Tomi Mäkelä (University of Helsinki, Finland) and have been described previously (23). ULK1/2 double knock-out MEFs were a kind gift from Craig Thompson (Memorial Sloan-Kettering Cancer Center) and have been described previously (24). TBK1/IKKϵ knock-out and matched MEFs were a kind gift from Shizuo Akira (Osaka University, Japan) and have been described previously (25). MEFs and 293T cells were grown in DMEM, supplemented with 10% fetal bovine serum and penicillin/streptomycin, and cultured at 37 °C, 5% CO_2_. For induction of autophagy, cells were typically grown to 75% confluency, washed twice, and incubated in Earle's balanced salt solution (EBSS) for 1 h (or complete medium as a control) unless indicated. MRT67307 (10 μm), MRT68921 (1 μm), AZD8055 (1 μm), or bafilomycin A1 (50 nm) was included where indicated. Transfection and transduction were as described (15).

##### Cell Lysis and Immunoprecipitation

Cell lysis and immunoprecipitation were carried out as described (15). Immunoblots were quantified by densitometry, using ImageJ.

##### Immunofluorescence

Immunofluorescence was carried out as described (26). Slides were visualized on a Nikon TiS inverted microscope, and images were processed using NIS Elements (Nikon) and Adobe Photoshop.

##### Live Cell Microscopy

Cells were washed and incubated in phenol red-free medium and imaged on a Nikon TiE inverted microscope with an Okolab environmental chamber at 37 °C, 5% CO_2_. Images were obtained and processed using NIS Elements (Nikon).

##### Kinase Assays

Initial ULK1 kinase assays were performed with GST-ULK1, produced in Sf9 cells, which is described on the MRC-PPU Reagents website. For other experiments, recombinant GST-ULK1 (wild type, kinase-dead (K46I), and M92T and M92Q) was expressed in 293T cells, purified, and eluted from a glutathione-Sepharose column. Kinase assays were carried out in 50 mm Tris-HCl, pH 7.4, 10 mm magnesium acetate, 0.1 mm EGTA, and 0.1% β-mercaptoethanol, containing 30 μm cold ATP, and 0.5 μCi of [γ-^32^P]ATP for 5 min at 25 °C. Prior to ATP addition, reaction mixes were prewarmed to 25 °C for 5 min. Reactions were stopped by the addition of sample buffer, followed by SDS-PAGE, transfer to nitrocellulose, and analysis by autoradiography and immunoblot. For IC_50_ curve measurements, kinase assays were performed as described (27), using myelin basic protein (Sigma) as a substrate. Kinase profiling was as described (28), performed by the International Centre for Kinase Profiling.

##### Statistical Analysis

Experiments were carried out a minimum of three times, and error bars represent S.E. Significance in related samples was determined by paired student's *t* test. A *p* value of 0.05 or less was deemed significant.

## RESULTS AND DISCUSSION

**FIGURE 1.**
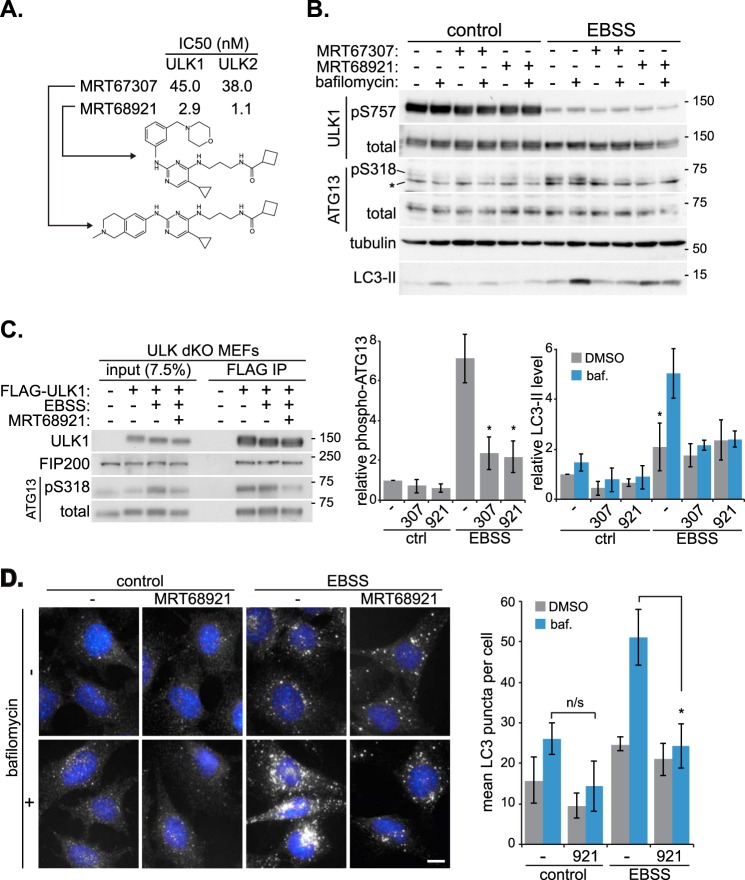
**MRT67307 and MRT68921 inhibit ULK and block autophagy in cells.** *A*, summary of MRT67307 and 68921 structures and *in vitro* ULK1 and ULK2 IC_50_ values. *B*, MEF cells were incubated in EBSS for 1 h in the presence or absence of 10 μm MRT67307, 1 μm MRT68921, or 50 nm bafilomycin A1, followed by lysis and immunoblotting with the indicated antibodies (* indicates nonspecific staining). Quantitation of phospho-serine 318 (*pS318*) of ATG13, normalized to total ATG13, is shown below next to quantitation of LC3-II levels, normalized to tubulin. *pS757*, phospho-Ser-757; *DMSO*, dimethyl sulfoxide; *ctrl*, control. *C*, representative blot of a FLAG immunoprecipitation from ULK1/2 double knock-out MEFs (*ULK dKO*) expressing FLAG-ULK1. *D*, representative micrographs of endogenous LC3 immunofluorescence in MEFs incubated in complete medium (*control*) or EBSS for 1 h in the presence or absence of 1 μm MRT68921 and 50 nm bafilomycin A1. Quantitation of LC3 puncta is shown on the *right*. All quantitation represent means ± S.E. from at least three independent experiments. *, *p* < 0.05. *Scale bar*, 10 μm. *n/s*, not significant.

*In vitro* screening of known kinase inhibitors led to the discovery that the TBK1 inhibitor, MRT67307 (29), also targeted ULK1 and ULK2 with high potency (IC_50_ values of 45 and 38 nm, respectively, Fig. 1*A*). It is of no surprise that a compound would target both ULK1 and ULK2, given the high degree of similarity between the kinase domains; it is also desirable given the degree of redundancy observed in knock-out mouse models (20). To find more potent inhibitors, we re-analyzed a closely related series of analogues generated during the original TBK1 screen. This led to the discovery of MRT68921 as the most potent inhibitor of both ULK1 and ULK2, with greater than a 15-fold reduction in the IC_50_ for ULK1 (2.9 nm) and greater than a 30-fold reduction for ULK2 (1.1 nm) (Fig. 1*A*). To see whether *in vitro* activity of the compounds could translate to inhibition in cells, we treated MEFs with either MRT67307 or MRT68921, in combination with amino acid withdrawal, to inhibit mTOR and activate ULK1. Bafilomycin A1 was also included to inhibit lysosomal turnover and enable autophagic flux measurement (30) (Fig. 1*B*). Loss of mTOR-mediated ULK1 phosphorylation occurred upon incubation of cells in EBSS, and this was not prevented by treatment with either of the MRT compounds. ATG13 phosphorylation at serine 318 was used as a measure of ULK1 activity (31), and this correlated well with EBSS treatment and loss of the ULK1 mTOR phosphorylation, showing greater than a 5-fold increase over basal levels (Fig. 1*B*). 10 μm MRT67307 was sufficient to reduce phospho-ATG13 to control levels, and in line with the *in vitro* IC_50_ values, 10-fold less MRT68921 (1 μm) resulted in a similar reduction. Therefore, these compounds can reduce ULK1 activity in cells. Importantly, these compounds also block autophagy as indicated by LC3-II levels. Basal autophagy was low under the experimental conditions used; however, EBSS treatment resulted in a 5-fold increase in bafilomycin-sensitive LC3-II levels, indicating strong mTOR-dependent autophagy induction. Both compounds blocked any bafilomycin-induced increase, demonstrating inhibition of autophagic flux. Both compounds behaved similarly, and although there was no LC3-II flux, a small 2-fold increase in LC3-II levels was observed.

ULK1 is part of a multi-protein complex, and at least two of these proteins, ATG13 and FIP200, are required for maximal ULK activity (13–15); it is possible that the MRT compounds inhibit ULK by disrupting these interactions. To look at this, we stably expressed FLAG-tagged ULK1 in ULK1 and ULK2 double-knock-out (dKO) cells and immunoprecipitated ULK in the presence or absence of autophagy-inducing conditions. As can be seen in Fig. 1*C*, the presence of inhibitor did not reduce the amounts of ATG13 and FIP200 co-precipitating with ULK1, but did reduce the level of phosphorylated ATG13 in this complex. To further assess autophagy, we looked at endogenous LC3 by immunofluorescence (Fig. 1*D*). Under control conditions, MRT68921 caused a slight reduction in basal LC3 puncta, indicating that the compound can block basal autophagy. However, the changes were not statistically significant, which could be due to low basal level of autophagy during the treatment. In contrast and as with the Western blot data, EBSS caused a large increase in bafilomycin-sensitive LC3 puncta accumulation. Likewise, this was blocked by both MRT compounds (although only MRT68921 is shown in Fig. 1*D*), confirming that these compounds do indeed block autophagy in cells. Also, like the Western blot data (Fig. 1*B*), a small 2-fold increase in distinct LC3 puncta was observed with the inhibitors, which may represent stalled phagophores (see below).

**FIGURE 2.**
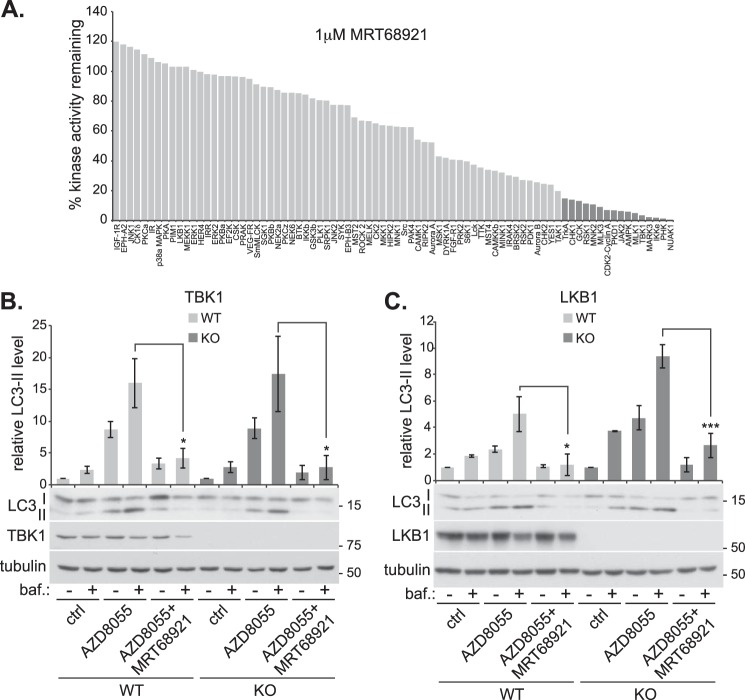
**Kinase selectivity of MRT68921.** *A*, activity of 80 recombinant kinases measured in the presence of 1 μm MRT68921. Data represent percentage of activity remaining relative to activity in the absence of inhibitor. *Darker shaded bars* to the *right* indicate kinases that were inhibited by over 80%. *B*, TBK1 knock-out (*KO*) and matched wild-type (*WT*) MEFs were incubated with/without 1 μm AZD8055 in the presence or absence of 50 nm bafilomycin (*baf.*) and 1 μm MRT68921. A representative immunoblot is shown with quantitation of LC3-II levels normalized to tubulin above. *ctrl*, control. *C*, similar to *panel B*, but LKB1 knock-out and matched wild-type MEFs were used. All quantitations are from three independent experiments ± S.E. *, *p* < 0.05, ***, *p* < 0.001.

Both MRT compounds inhibit ULK1 *in vitro* and in cells, and also block autophagy. However, they could be targeting autophagy through another kinase. The *in vitro* kinase profiling of MRT67307 revealed that it is a relatively specific kinase inhibitor, targeting TBK1/IKKϵ but also hitting the AMPK-related kinases (33). To assess the specificity of MRT68921, we profiled the inhibitor at 1 μm against a broad panel of 80 protein kinases representing all areas of the human kinome (Fig. 2*A*). As with MRT67307, MRT68921 was relatively specific, but still inhibited a number of kinases by over 80% (Fig. 2*A*, *dark shaded bars*). Most notably, TBK1/IKKϵ and the AMPK-related kinases were still targeted. TBK1 and AMPK have been implicated in autophagy (34–40), so it was important to rule these out as autophagy-inhibiting targets of MRT68921. To analyze the role of TBK1, we took knock-out MEFs and asked whether autophagy could still be induced and inhibited by MRT68921 (Fig. 2*B*). To induce autophagy, we utilized the mTOR inhibitor AZD8055 (41) and in TBK1-containing cells, we found a large increase in LC3-II flux, which was inhibited by MRT68921. In TBK1 knock-out cells, the pattern of LC3-II accumulation was almost identical, indicating that TBK1 is not essential for mTOR-mediated autophagic flux and is not the autophagy-inhibiting target of MRT68921. Of course we do not rule out the involvement of TBK1 in more specific forms of autophagy such as basal autophagy or xenophagy. However, this result highlights the caution that is needed when using TBK1 inhibitors to validate a role for TBK1 in autophagy.

To analyze AMPK-related kinase involvement, we took advantage of the fact that these kinases, with the exception of AMPK (which can also be activated by CaMKK2), require LKB1-mediated phosphorylation for activation (42). Using LKB1 knock-out MEFs, we found that LC3 flux was comparable with matched, wild-type MEFs and was inhibited to the same extent with MRT68921 (Fig. 2*C*). Therefore, these kinases are not the target of MRT68921 in blocking autophagy. Interestingly, LC3 flux was slightly higher under basal and autophagy-inducing conditions in the LKB1 knock-out MEFs, which is similar to that seen in AMPK knock-out cells (36). This supports the idea that AMPK, or one of the related kinases, although not essential for autophagy, does play a regulatory role.

**FIGURE 3.**
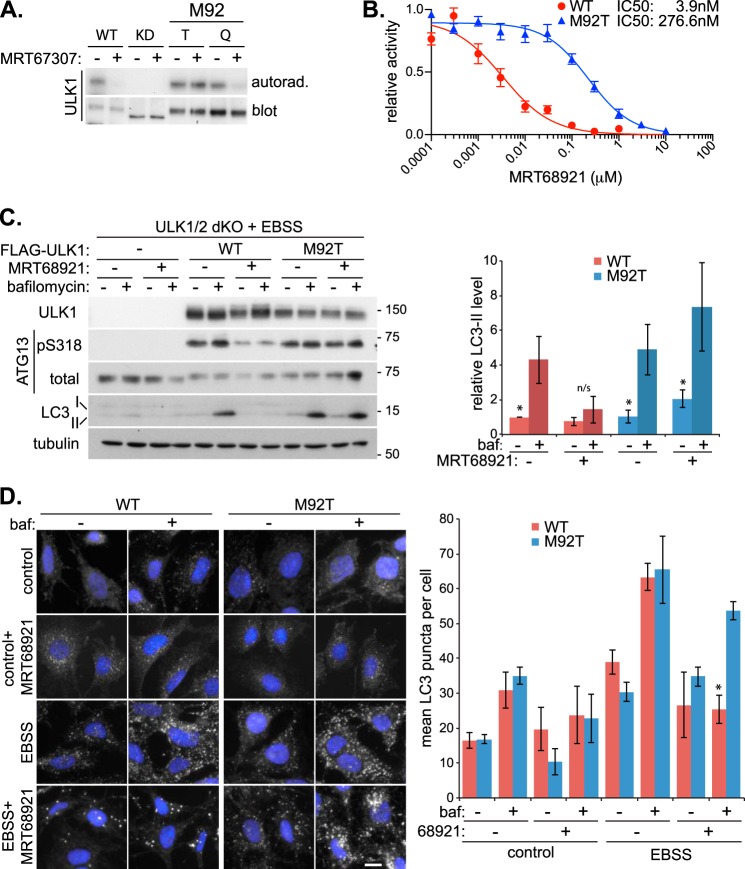
**MRT68921 specifically blocks autophagic flux through ULK1 inhibition.** *A*, *in vitro* ULK1 autophosphorylation kinase assay in the presence of 1 μm MRT67307 with GST-tagged wild-type (WT), kinase-dead (*KD* (K46I)), and M92T (*T*) and M92Q (*Q*) ULK1. *B*, *in vitro* IC_50_ plot with GST-tagged WT and M92T ULK1 in the presence of MRT68921. *C*, ULK1/2 dKO MEFs, stably expressing FLAG-tagged WT or M92T ULK1, were treated with EBSS for 1 h in the presence or absence of 1 μm MRT68921 and 50 nm bafilomycin (*baf*). Lysates were subjected to immunoblotting with the indicated antibodies. Quantitation of LC3-II levels, normalized to tubulin, is shown on the *right. pS318*, phospho-serine 318. *D*, endogenous LC3 immunofluorescence in ULK dKO MEFs rescued with the indicated FLAG-tagged ULK1 construct. Cells were incubated in complete medium (*control*) or EBSS for 1 h in the presence or absence of 1 μm MRT68921 and 50 nm bafilomycin. Quantitation of LC3 puncta is shown on the *right*. All quantitations represent means ± S.E. from at least three independent experiments. *, *p* < 0.05. *Scale bar*, 10 μm. *n/s*, not significant.

Both the MRT compounds are competitive ATP-binding site inhibitors and may inhibit autophagy through other kinases, or even non-kinase ATP-binding proteins. To rule this out and confirm ULK1 as the target, we generated a drug-resistant ULK1 mutant. The gatekeeper residue is a bulky hydrophobic amino acid present at the base of the ATP-binding pocket in all typical protein kinases. When this residue is mutated, it can change the ATP binding properties of the kinase. To identify residues that might result in loss of inhibitor binding, but retain ATP binding, we mutated the gatekeeper residue of ULK1 (methionine 92) to various amino acids. Mutation to alanine or glycine resulted in loss of kinase activity, but a change to threonine or glutamine resulted in an ULK1 construct that retained *in vitro* activity in a simple autophosphorylation kinase assay (Fig. 3*A*). Satisfyingly, mutation of methionine 92 to threonine (M92T) allowed ULK1 to retain kinase activity in the presence of MRT67307, at a concentration that inhibited WT and the glutamine mutant. To analyze the resistance of ULK1 M92T to the MRT compounds in greater detail, we characterized the IC_50_ for this mutant and found over a 70-fold decrease in sensitivity, confirming the drug resistance (Fig. 3*B*).

We next confirmed that the M92T mutant could rescue the MRT68921-mediated autophagy inhibition. For this, either WT or M92T FLAG-ULK1 was stably expressed in ULK1/2 dKO MEFs (Fig. 3*C*). Re-expression of WT or M92T ULK1 restored ATG13 phosphorylation and autophagy (as observed by LC3-II flux), caused by the loss of endogenous ULK1 and ULK2. In these cells, it appears that ULK1 is sufficient to rescue autophagy, highlighting the redundancy of ULK2. Importantly, MRT68921 was able to inhibit the WT-restored ATG13 phosphorylation and autophagy similarly to cells expressing endogenous ULK1 (compare Fig. 1*B* with 3C). However, in cells expressing a similar level of M92T ULK1, MRT68921 failed to reduce either ATG13 phosphorylation or LC3 flux (Fig. 3*C*). This indicates that MRT68921 is indeed blocking autophagy through ULK1 kinase inhibition. To our knowledge, this is the first example of specifically inhibiting ULK1 with a small molecule to disrupt autophagy.

**FIGURE 4.**
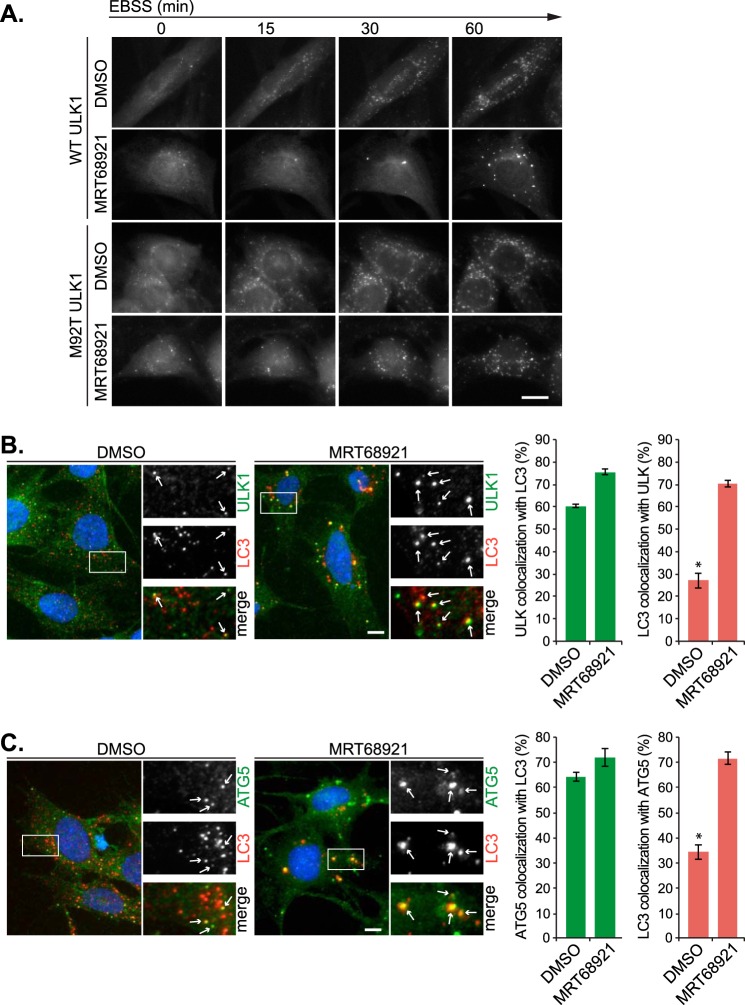
**ULK inhibition also disrupts autophagosome maturation.** *A*, ULK1 rescue MEFs stably expressing GFP-LC3 were washed and placed in complete medium or EBSS with/without 1 μm MRT68921, and images were taken every 30 s for 90 min. Representative images are shown from EBSS-treated samples, derived from supplemental Movie 1 (WT ULK, *top panels*) or supplemental Movie 2 (M92T ULK1, *bottom panels*). *DMSO*, dimethyl sulfoxide. *B*, MEF cells were incubated in EBSS for 1 h with/without 1 μm MRT68921 followed by fixing and staining with antibodies to LC3 (*red*) or ULK1 (*green*). Quantitation of the degree of co-localization is shown to the *right. C*, cells, treated as in *panel B*, were stained for LC3 (*red*) and ATG5 (*green*). All quantitations represent means ± S.E. from at least three independent experiments. *, *p* < 0.05. *Scale bars*, 10 μm.

To confirm inhibition is through ULK1, we used the ULK1 rescue MEFs to analyze LC3 immunofluorescence (Fig. 3*D*). Cells expressing both WT and M92T ULK1 displayed a comparable increase in the number of bafilomycin-sensitive LC3 puncta in response to EBSS treatment. Similarly, the LC3 puncta flux was blocked by MRT68921 treatment only in the WT ULK1-expressing cells, not the M92T ULK1 cells, confirming the MRT compound blocks autophagy through ULK1 inhibition. As with the immunofluorescence analysis in cells endogenously expressing ULK (Fig. 1*D*), we did see a small increase in the formation of distinct and bright LC3 puncta upon MRT68921 treatment in the WT-expressing cells, and given that no LC3 flux was observed, these structures could possibly represent stalled phagophores or immature autophagosomes. These bright puncta were not readily observed under control conditions with inhibitor, indicating that stimulated autophagy differs somewhat from basal autophagy or that the equivalent puncta that accumulate are smaller and harder to distinguish from the background staining. To gain further insight into these aberrant structures, we carried out live cell microscopy in GFP-LC3-expressing ULK1 rescue cells (see supplemental Movies 1 and 2). Incubation of both WT-expressing and M92T ULK1-expressing cells in EBSS resulted in rapid formation of GFP-LC3 autophagosomes after as little as 15 min. In contrast, in WT but not M92T cells, MRT68921 treatment delayed the formation of GFP-LC3 structures, which only started to accumulate following 30 min of treatment. Additionally, these structures appeared brighter and morphologically distinct to those formed in the untreated WT cells (or treated/untreated M92T ULK1 cells). The reader is encouraged to watch the movies, but representative images are shown in Fig. 4*A*. The distinct appearance, timing, and lack of flux of the inhibitor-induced LC3 structures suggested that they are not regular autophagosomes. We hypothesized that ULK1 inhibition primarily blocks autophagy induction; however, some residual ULK activity remains to allow autophagosome initiation but not maturation. To analyze this further, we co-stained cells for LC3 and the phagophore markers ULK1 and ATG5 (Fig. 4, *B* and *C*). We found that the majority of the inhibitor-induced LC3 structures, over 70%, contained ULK1 and ATG5, which is in contrast to structures found in the absence of inhibitor that showed less than a third co-localizing with ULK1 or ATG5. Additionally, inhibitor treatment did not appear to reduce the number of ULK1 or ATG5 puncta forming upon autophagy stimulation. Firstly, this suggests that ULK activity is not essential for its recruitment to autophagosomal structures (a similar observation has been made with kinase-dead ULK1 constructs (43)). Secondly, given that the LC3 structures contain ULK1 and ATG5, both phagophore markers (44, 45), this suggests that ULK1 plays a role in phagophore maturation into complete autophagosomes. This is not an unreasonable proposal as a similar phenomenon has been observed in yeast, whereby expression of a kinase-dead form of Atg1 (yeast ULK) did indeed block autophagy, but also resulted in increased recruitment of Atg8 (yeast LC3 homolog) and Atg17 to the pre-autophagosomal structure (46). Additionally, in mammalian cells, dissociation of ULK1 from the omegasome precedes autophagosome maturation (47). Our data fit well with these observations, suggesting a function for Atg1/ULK1 activity in disassembly of the pre-autophagosomal structure/phagophore to allow autophagosome maturation. However, further work in characterizing these structures is needed to clarify this. We therefore suggest that ULK regulates autophagy through at least two mechanisms: (i) regulation of the VPS34 complex, by AMBRA1/Beclin1 phosphorylation, upstream of LC3 conjugation (12, 32); and (ii) controlling maturation of the phagophore and transition to the mature autophagosome, downstream of LC3 conjugation.

In summary, we have shown that MRT67307, and especially MRT68921, potently inhibit ULK1 and ULK2 *in vitro* and ULK1 in cells. Importantly, this was sufficient to block autophagic flux. We demonstrated specificity in the autophagy block through the generation of the drug-resistant M92T ULK1 mutant. Although it might be expected that pharmacologically inhibiting ULK1 would block autophagy, this has not been previously shown and highlights the importance of ULK kinase activity in autophagy induction. It also acts as a proof-of-principle in developing small molecule inhibitors to block autophagy for therapy in diseases such as cancer. Finally, we have developed a system to enable molecular analysis of ULK1 function in autophagy and revealed a role for ULK1 in the maturation of autophagosomes. Future work will hopefully identify the targets of ULK1 that enable the phagophore to autophagosome switch.

## Supplementary Material

Supplemental Data

## Footnotes

2 The abbreviations used are:

mTOR: mammalian target of rapamycin
EBSS: Earle's balanced salt solution
MEF: mouse embryonic fibroblast
dKO: double-knockout
ULK: Unc51-like kinase
TBK1: TANK-binding kinase 1
TANK: TRAF family member-associated NF-κB activator
TRAF: tumor necrosis factor receptor-associated factor
LKB1: liver kinase B1
AMPK: AMP-activated protein kinase
IKK: IκB kinases
LC3: light chain 3.
