# Supplementary material for: Pharmacological Inhibition of ULK1 Kinase Blocks Mammalian Target of Rapamycin (mTOR)-dependent Autophagy
Source: J Biol Chem. 2015 Apr 1;290(18):11376–83. doi: 10.1074/jbc.C114.627778 (PMC4416842; doi:10.1074/jbc.C114.627778)
Supplement: Supplemental Data [file supp_290_18_11376__index.html]

Pharmacological Inhibition of ULK1 Blocks mTOR-Dependent Autophagy — Pharmacological Inhibition of ULK1 Kinase Blocks Mammalian Target of Rapamycin (mTOR)-dependent Autophagy — REPORT: ULK1 Inhibitor — Supplemental Data 

# Pharmacological Inhibition of ULK1 Kinase Blocks Mammalian Target of Rapamycin (mTOR)-dependent Autophagy

## Supplemental Data

**Files in this Data Supplement:**

- Movie1 (.mov, 54.8 MB) - Movie1. GFP-LC3 expressing WT-ULK1 rescue MEFs were incubated in complete media (ctrl) or EBSS with/without 1&#x26;#956;M MRT68921 and imaged every 30sec. Scale bar, 10&#x26;#956;m.
- Movie2 (.mov, 33.8 MB) - Movie2. GFP-LC3 expressing M92T-ULK1 rescue MEFs were incubated in complete media (ctrl) or EBSS with/without 1&#x26;#956;M MRT68921 and imaged every 30sec. Scale bar, 10&#x26;#956;m.
